# Making shiny objects illuminating: the promise and challenges of large language models in U.S. health systems

**DOI:** 10.1038/s44401-025-00014-7

**Published:** 2025-03-18

**Authors:** Rui Zhang, James Zou, Ashley N. Beecy, Yiye Zhang, Jiang Bian, Genevieve B. Melton, Cui Tao

**Affiliations:** 1 Department of Surgery and Center for Learning Health System Sciences, University of Minnesota, Minneapolis, MN USA; 2 Institute for Health Informatics, University of Minnesota, Minneapolis, MN USA; 3 Department of Biomedical Data Science, Stanford University, Stanford, CA USA; 4 Department of Medicine, Division of Cardiology, Weill Cornell Medicine, New York, NY USA; 5 Information Technology, NewYork-Presbyterian Hospital, New York, NY USA; 6 Department of Population Health Sciences, Weill Cornell Medicine New York, New York, NY USA; 7 Biostatistics and Health Data Science, School of Medicine, Indiana University, Indianapolis, IN USA; 8 Regenstrief Institute, Indianapolis, IN USA; 9 Department of Artificial Intelligence and Informatics, Mayo Clinic, Jacksonville, FL USA

The rapid advancement of generative artificial intelligence (AI), especially large language models (LLMs) in recent years, has transformed the AI industry across society and in particular for the academic biomedical and health communities. Many powerful LLMs, such as GPT-4o from OpenAI and Claude from Anthropic, are proprietary or closed models with restricted access to their architecture, training data, and training processes. On the other hand, open-source LLMs, such as LLaMA^1^ from Meta and the most recently released DeepSeek^2^ funded from the High-Flyer hedge fund, allow public access to their code accompanied by scientific publications describing their structure and training processes. This fosters transparency and collaboration, enabling broader research and translation in AI. Given the critical importance of patient privacy and data security in healthcare, powerful open-source LLMs are uniquely positioned to drive the development of domain-specific LLM solutions, with additional training with healthcare data^3^. However, despite the transformative potential, a number of key barriers remain, particularly regarding cybersecurity and health system resource planning. These challenges are further complicated by the uncertainty and chaos around the current administration’s directions towards AI, healthcare delivery, and scientific research. After all, many health systems were still adapting to traditional AI, which was only beginning to be implemented in select settings with emerging impacts when ChatGPT took society by storm^4^. Are health systems ready for the next shiny objects - generative AI and LLMs? As part of the “Generative AI in Health Systems – Development, Implementation, and Evaluation” collection, this editorial explores and poses a question: How should researchers, innovators, and healthcare systems leaders grapple with the changing and intertwining landscapes of LLMs to harness its full potential? While we focus on U.S. health systems in this editorial, we welcome global perspectives in the collection.

## AI governance for safe and effective use of generative AI

Many health systems are currently navigating the complexities of AI integration, spanning traditional predictive models, computer vision-based diagnostic tools, and LLMs^3^. Generative AI solutions, in particular, require careful navigation of the regulatory landscape to ensure compliance and alignment with established guidelines as well as new governance considerations. Effective management requires a thorough understanding of the evolving regulatory and legal requirements of incorporating AI in healthcare, as well as risk management strategies. These regulations include the Health Insurance Portability and Accountability Act (HIPAA) and the Office for Civil Rights (OCR) Section 1557 of the Affordable Care Act (ACA), among other existing clinical regulations. However, the regulatory landscape of AI in the U.S. has been chaotic in recent days, as reflected by the rescission of the Biden administration’s executive order on AI safety^5^, the uncertainty around many public agencies including U.S. Food and Drug Administration (FDA) and National Institute of Standards and Technology (NIST)^6^ which created the AI Risk Management Framework (RMF). While there are consortiums, such as Coalition for Health AI (CHAI) and Health AI Partnership (HAIP), recommendations from these organizations do not have mandatory powers and may offer idealistic recommendations beyond practicality. In the meantime, the heightened demand for AI is increasingly forcing health systems to set up structures to provide ad hoc local interpretations on regulatory considerations, data use, and clinical impact.

In the last few years, these structures have come to be referred to as *AI governance*, an emerging and essential aspect of the introduction of AI to healthcare^7,8^. AI governance thus far has been led by various roles around data and digital health, some existing and some newly established in health systems^8^. It reflects a health system’s culture and critically influences how patient care is changed by AI. AI governance is broader than merely complying with regulations, but also includes establishing realistic processes within health systems around intake, review, implementation, and monitoring. Compared to traditional predictive models that are increasingly implemented in healthcare^9,10^, LLMs dramatically complicates this process due to its non-deterministic nature, tendency for hallucination, and non-comprehensiveness^11^. From an AI governance perspective, the proprietary models pose challenges on transparency and secondary use of data. On the other hand, their open-source counterparts offer deeper interpretability through open access and community contributions, and more transparent safety evaluations fit for health systems’ needs. Below, we discuss three critical areas for integrating LLMs in health systems: 1) data infrastructure and cybersecurity, 2) resource planning, and 3) translation of research.

## Data infrastructure and cybersecurity

The integration of LLMs within health systems requires substantial and advanced resources, support, and expertise to maintain and optimize, including scalable high-performance computing hardware and sustainable computational environments. Whereas traditional AI systems were often designed for batch processing or offline analysis with fewer real-time interaction demands, LLMs - depending on the use case - require infrastructure optimized for low-latency inference and seamless compatibility with interoperability standards such as Fast Healthcare Interoperability Resources (FHIR) to ensure fast response times in clinical workflows. LLM models could also be sensitive to changes in EHR practices, which adds maintenance complexity and hinders widespread adoption. Variations in data entry protocols, coding standards, and documentation practices across institutions can lead to model performance degradation over time if not regularly updated and retrained. High-quality, unbiased, and domain-specific health data is a key factor in ensuring the effective adaptation of open-domain LLMs. Ensuring diverse and inclusive training data, representative of different populations, health systems, and clinical environments, is essential to mitigate these risks. Additionally, having a structure for continuous monitoring and bias auditing post-deployment are necessary to identify and address emerging biases as healthcare practices evolve.

Tying closely with AI and data governance, data privacy and security are paramount due to the sensitive nature of patient information. Ensuring the confidentiality, integrity, and availability of health data is critical, especially when LLMs are involved in processing large volumes of protected health information (PHI) and is essential to safeguard against unauthorized access and potential breaches. As LLMs become more integrated into health IT infrastructure, cybersecurity must be a top priority. Open-source LLMs offer several advantages but also present unique security challenges and uncertainties. One key benefit is to host models on-premises, allowing organizations to maintain greater control over security measures. Their transparency and adaptability make them particularly appealing for institutions seeking to use applied AI in healthcare. Some believe open-source LLMs can be deployed locally behind institutional firewalls, ensuring transparent and controllable security profiles compared to transmitting PHI to external API endpoints. On the other hand, open-source LLMs present unique security challenges, such as the potential vulnerabilities in the codebase that could be exploited if not regularly audited and updated. Robust cybersecurity measures—such as advanced encryption protocols, secure access controls, continuous threat monitoring, and regular security audits—are essential to protect AI solutions, the sensitive health data they process, and risks to healthcare organizations. Some even hypothesized that the LLMs themselves, especially DeepSeek, may contain “malicious” actions. For example, the news media has alleged that DeepSeek’s answers include Chinese propaganda^12^. Putting national politics aside, LLMs—including U.S.-based open-source models like Llama and even commercial LLMs, including ChatGPT—are known to hallucinate and generate misinformation. If so, traditional security controls would be insufficient, requiring the development of new and innovative safeguards.

## Cost structure and resource planning

The economics of AI, and particularly LLMs, is a crucial consideration for health systems when considering long-term sustainability and reimbursement^13^. Developing and deploying generative AI systems in healthcare involves significant investments in data collection, model development, computational resources, software tools, and ongoing maintenance. Once embedded in health systems, specialized health IT teams—and clinical oversight—are needed to manage complex AI and automation workflows, supporting AI models such as LLMs and other generative AI solutions from multiple angles including AI governance, review, deployment, and cybersecurity^14^. These roles demand ongoing skill enhancement across job levels and specialized technical training in AI model development, optimization, and deployment^15^. LLMs have applications across diverse domains, and health systems will likely face increasing procurement demands as vendors integrate these technologies. A notable advantage of open-source LLMs is the flexibility they offer in deployment options. Proprietary models typically require cloud-based subscription and per-usage services which can be costly and subject to outside infrastructure and management services. Open-source LLMs offer a more customizable alternative, enabling greater flexibility in deployment across an organization’s preferred infrastructure, whether on-premises or in the cloud. Going beyond the hype and public relations, a careful evaluation of the costs and clinical impact of generative AI will eventually determine its future in health systems. At a national level, the consideration of cost structure and resource planning should also include the gap between health systems that have the resources vs. those that do not, and the consequential impact faced by patients. Similarly, with generative AI, the gap between scientific research and industry development has been wider than ever^16^. We need national-level conversations on the integration of research-based generative vs. vendor products in health systems, and how to ensure that translation of scientific research on generative AI to patient care is not discouraged in the process.

## Translation of research and innovation to the bedside

Health systems play a crucial role in providing venues for research on LLM implementation, evaluating user experience, incorporating reinforcement learning mechanisms, and developing analytics to support longer term monitoring. We have begun to see LLMs in actual use in health systems, such as Epic’s MyChart In-Basket Augmented Response Technology (ART)^17^ and various medical summarization tools^18,19^. One of the most promising applications in healthcare lies in its ability to enhance clinical decision making through processing vast amounts of multimodal electronic health records (EHRs) data, including structured and coded EHRs, imaging, genetics, and clinical notes. These LLMs can be fine-tuned using local health system data to create domain-specific and task-driven models. This customization allows open-source LLM to adapt to different healthcare settings, enhancing its effectiveness in various regions or populations. For example, in the precision oncology domain, the open-source Qwen-1.5 14B model was trained on local EHR data from cancer patients to generate OncoLLM^20^ which outperformed other close-source LLMs such as GPT-3.5 for clinical trials matching. Similarly, CancerLLM^21^ was built upon Mistral 7B which was further trained on local clinical records for cancer phenotyping extraction and diagnosis generation tasks, and demonstrated efficiency and robustness compared with other LLMs.

Despite its potential, integrating open-source LLM or AI models into health systems and clinical research presents significant opportunities alongside notable challenges. A critical limitation in current AI development processes is the insufficient incorporation of human intelligence, particularly from clinicians who provide nuanced insights to data interpretation and decision-making. AI-driven approaches can miss the contextual nuanced understanding that clinicians bring to accurate and ethical data interpretation and clinical decision-making^19^. As is the case with traditional AI, incorporating clinicians in the development and feedback cycles of clinical applications of LLMs enhances model relevance and ensures better alignment with clinical realities. Additionally, integrating LLMs into healthcare workflows necessitates dedicated efforts to train clinicians training and promote their acceptance to mitigate resistance and optimize human-AI interactions. Establishing multidisciplinary teams comprising data scientists, IT experts, clinicians, ethicists, and healthcare administrators can facilitate the development of local LLM solutions that are clinically relevant, ethically sound, and operationally feasible. Additionally, the complexities of AI governance, coupled with evolving research oversight, such as Institutional Review Board (IRB) evaluations, are likely to introduce delays in translating LLM research into clinical practice. This delay potentially will widen the gap between research-based and industry-made LLMs, biasing towards LLMs with more financial value.

## Recommendations

Even for health systems experienced with AI, introduction of LLMs into routine care will require non-trivial learning curves. Establishing flexible yet robust evaluation frameworks will guide the development and deployment of LLMs within health systems, ensuring both data privacy and adherence to ethical standards. Collaborative efforts to develop large, diverse, and interoperable datasets can enhance model robustness and reduce the risk of performance variability. We need success stories, but also lessons learned from failures, for health systems leaders, innovators, and researchers to develop, critically evaluate and improve healthcare delivery in the era of rapidly evolving AI techniques. The considerations raised above highlight the importance of future efforts to address technical and ethical challenges, ultimately aiming to maximize the positive impact of LLM and similar AI technologies on healthcare systems. By fostering a comprehensive grounded approach, one that balances robust health IT infrastructure, adaptability to local systems, thorough validation of generalizability, and meaningful human-in-the-loop integration, health systems can harness the transformative power of LLMs. This strategy not only enhances the reliability and security of AI applications but also safeguards patient welfare and promotes health across diverse healthcare environments.
